# Association between vancomycin therapeutic drug monitoring and improved clinical outcomes in critically ill patients receiving renal replacement therapy: a retrospective cohort study

**DOI:** 10.3389/fphar.2025.1715023

**Published:** 2026-01-07

**Authors:** Huaidong Peng, Xiaomei Tang, Lijin Chen, Qilin Yang, Jinliang Cai, Tingting Xie, Dan Xu

**Affiliations:** 1 Department of Pharmacy, The Second Affiliated Hospital, Guangzhou Medical University, Guangzhou, China; 2 Department of Neurology, Guangdong Sanjiu Brain Hospital, Guangzhou, China; 3 Department of Critical Care, The Second Affiliated Hospital, Guangzhou Medical University, Guangzhou, China; 4 Department of Critical Care, Gaoming District People’s Hospital of Foshan City, Foshan, China; 5 Department of Gastroenterology, The Second Affiliated Hospital, Guangzhou Medical University, Guangzhou, China

**Keywords:** vancomycin, therapeutic drug monitoring, critically ill patients, renal replacement therapy, mortality

## Abstract

**Introduction:**

Vancomycin is commonly prescribed for serious infections in critically ill patients. A substantial proportion of these individuals present with renal impairment or develop acute kidney injury (AKI), and some may require renal replacement therapy (RRT). Different RRT modalities can substantially affect vancomycin pharmacokinetics, thereby posing challenges for individualized dosing. Although therapeutic drug monitoring (TDM) is recommended by clinical guidelines to optimize drug exposure, its association with mortality among critically ill patients undergoing RRT has not been well characterized.

**Methods:**

This retrospective study used the MIMIC-IV database to identify adults with an initial ICU admission who received RRT within the first week and intravenous vancomycin during the ICU stay. Patients were classified into TDM and non-TDM groups according to whether TDM was performed. The primary outcome was 30-day all-cause mortality. To control for confounding, baseline characteristics were balanced using inverse probability of treatment weighting (IPTW) based on propensity scores. Associations between TDM and mortality were assessed using IPTW-weighted Cox regression, with results compared to unweighted Cox models. Subgroup analyses stratified by clinical characteristics and RRT modalities were performed to explore effect heterogeneity. Sensitivity analyses addressing missing cumulative vancomycin dose were conducted using random forest imputation, complete-case analysis, and the missing-indicator method.

**Results:**

A total of 2,085 patients were included, with 1,556 in the TDM group and 529 in the non-TDM group. 30-day mortality was significantly lower in the TDM group (38.9% vs. 48.8%, *P* < 0.001). Multivariable Cox regression analyses, both before and after IPTW adjustment, demonstrated a consistent association between TDM and reduced mortality risk (hazard ratio [HR] 0.457–0.478, all *P* < 0.001). Kaplan–Meier analysis further confirmed higher survival in the TDM group (log-rank *P* < 0.001). In the continuous renal replacement therapy (CRRT) subgroup, all models yielded consistent results, with TDM associated with significantly lower mortality (HR 0.427–0.431, all *P* < 0.001). Sensitivity analyses supported the robustness of these findings, as the inverse association between TDM and mortality persisted across all approaches to handling missing vancomycin dose data (HR 0.474–0.610, all *P* < 0.001).

**Conclusion:**

Vancomycin TDM was significantly associated with reduced 30-day mortality in critically ill patients receiving RRT, with an even stronger effect observed in those undergoing CRRT. These findings support the potential clinical relevance of TDM in this high-risk population.

## 1 Introduction

Vancomycin is widely recognized as a first-line antibiotic in intensive care units (ICUs) for the treatment of severe Gram-positive bacterial infections, particularly those caused by methicillin-resistant *Staphylococcus aureus* (MRSA) (Purja et al., 2024; Rybak et al., 2020; Mehta et al., 2020). However, its clinical use remains challenging due to a narrow therapeutic window and pronounced interindividual pharmacokinetic variability (Rybak et al., 2020; Álvarez et al., 2016). Critically ill patients often exhibit complex pathophysiological changes, including advanced age, abnormal body weight, hypoalbuminemia, and multiple organ dysfunction, which markedly alter vancomycin pharmacokinetics. (Tanaka, 2025; Tesfamariam et al., 2024; Idasiak-Piechocka et al., 2025). These alterations may lead to substantial fluctuations in serum drug concentrations, complicating individualized dosing strategies and increasing the risk of treatment failure or drug-related toxicity (Ghasemiyeh et al., 2022).

Therapeutic drug monitoring (TDM) has been widely advocated to optimize vancomycin exposure and minimize nephrotoxicity, particularly in response to the dosing challenges encountered in critically ill patients (Rybak et al., 2020; He et al., 2020; Peng et al., 2024a). Currently, vancomycin TDM primarily utilizes two approaches: trough concentration monitoring and estimation of the area under the concentration–time curve (AUC) (Stewart et al., 2021; Liu et al., 2024). Trough monitoring is simple and feasible in most hospitals, making it particularly suitable for resource-limited settings (Yu et al., 2023). As an indirect measure of drug exposure, it has been widely used for over a decade (Reuter et al., 2022; Rybak et al., 2009). However, trough levels may not accurately reflect overall vancomycin exposure, potentially leading to inappropriate dosing, reduced efficacy, or increased toxicity (Yahav et al., 2019; Wan et al., 2018). In contrast, AUC estimation is considered a more precise pharmacokinetic parameter that better predicts therapeutic efficacy and nephrotoxicity, and has attracted growing attention in recent years (Rybak et al., 2020; Lim et al., 2023). Nonetheless, this approach requires advanced computational tools and clinical pharmacy support, which presents challenges to its widespread clinical implementation (Liu et al., 2024). In summary, while trough monitoring remains widely used due to its simplicity, AUC-guided TDM is increasingly promoted as a superior strategy (Liu et al., 2024; Jorgensen et al., 2023).

TDM is especially pertinent to patients undergoing renal replacement therapy (RRT), where vancomycin pharmacokinetics are further influenced by factors such as RRT modality (e.g., continuous renal replacement therapy [CRRT] or intermittent hemodialysis [IHD]), dialysis intensity, effluent flow rate, and residual renal function (Tsai et al., 2024; Lewis and Nolin, 2021; Smeets et al., 2023). These variables introduce substantial variability in drug clearance, leading to unpredictable exposure and potentially increasing the risk of therapeutic failure or toxicity. Although TDM has been associated with reduced adverse events and improved outcomes in some studies, most available evidence is limited to the general ICU population (Peng et al., 2024a; Peng et al., 2024b; Ye et al., 2013). High-quality data evaluating the survival benefits of TDM in patients receiving RRT, especially with respect to different RRT modalities, remain scarce. Further real-world evidence is needed to establish the clinical value of TDM and inform its routine application in this high-risk population. Recent advances in model-informed precision dosing (MIPD) have shown potential in optimizing vancomycin therapy (Matsumoto et al., 2022), particularly in patients receiving RRT (Oda et al., 2023). Tools such as InsightRX Nova (Insight Rx, 2021) now provide individualized dosing support for complex populations, including those on CRRT. MIPD is built upon the foundation of TDM but extends beyond traditional TDM by integrating advanced pharmacometric modeling and individualized patient data. Although such tools show promise, their real-world impact on patient outcomes requires further validation.

Accordingly, we conducted a retrospective cohort study using the large, publicly available MIMIC-IV database to evaluate the association between vancomycinTDM and 30-day all-cause mortality in critically ill patients receiving RRT. We also further explored whether the effect of TDM varied across RRT modalities, including CRRT, IHD, and peritoneal dialysis (PD).

## 2 Methods

### 2.1 Data source

The data for this study were obtained from the Medical Information Mart for Intensive Care IV (MIMIC-IV, version 3.1), a publicly available and fully de-identified database containing comprehensive health records of ICU patients admitted to the Beth Israel Deaconess Medical Center between 2008 and 2022 (Johnson et al., 2023; Johnson et al., 2024). It includes detailed information on demographics, vital signs, laboratory test results, comorbidities, treatments, and clinical outcomes, enabling comprehensive analyses of critically ill populations. One of the authors (Huaidong Peng) completed the NIH-required online course Protecting Human Research Participants (Certification No. 59679596) and was granted authorized access to the MIMIC-IV database for research purposes. All data are fully de-identified to protect patient privacy, and informed consent was therefore not required. This study was conducted in accordance with the Strengthening the Reporting of Observational Studies in Epidemiology (STROBE) guidelines and the ethical principles of the Declaration of Helsinki (von Elm et al., 2007). As the study utilized a fully de-identified, open-access dataset, ethical approval was waived by the Ethics Committee of the Second Affiliated Hospital of Guangzhou Medical University. The MIMIC-IV database is publicly available via the PhysioNet platform (https://physionet.org/).

### 2.2 Patient inclusion

**FIGURE 1:**
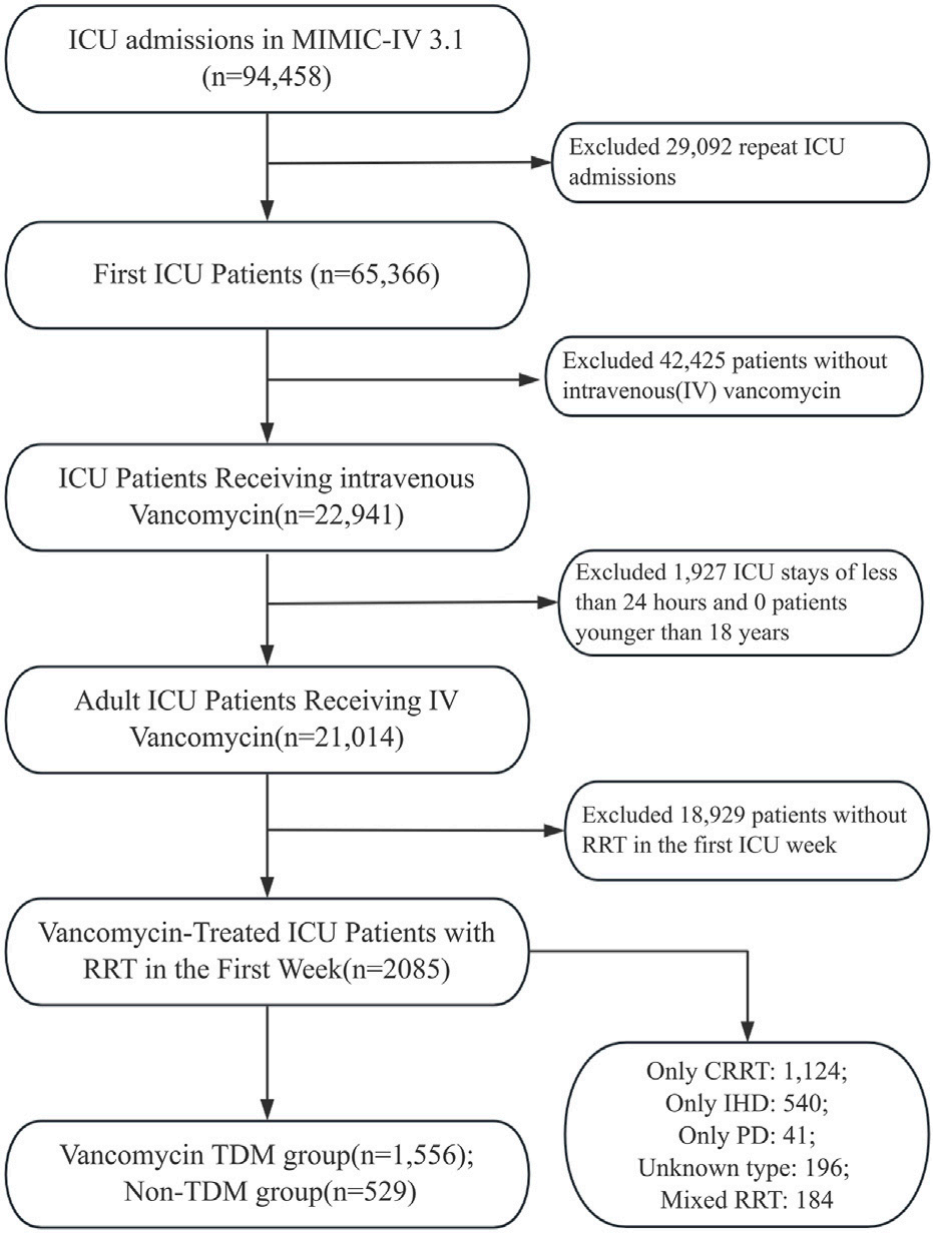
Flow chart depicting the enrollment process for patients.

ICU patients who received intravenous vancomycin therapy were included in this study. To maintain uniformity, only patients with their first ICU admission recorded in the MIMIC-IV database were considered. Additional inclusion criteria were: age ≥18 years, ICU length of stay ≥24 h, and receipt of RRT within the first 7 days of ICU admission. The exclusion criteria were as follows: (1) patients without a first ICU admission; (2) ICU length of stay <24 h; (3) patients younger than 18 years; (4) no documented intravenous vancomycin therapy; (5) did not receive RRT within the first 7 days of ICU admission. After applying these criteria, a total of 2,085 patients were included in the final cohort. The detailed patient selection process is illustrated in Figure 1.

### 2.3 Data extraction

We used Structured Query Language (SQL) to systematically extract patient data from the MIMIC-IV database. The extracted variables included demographics (age, sex, and race); vital signs (heart rate, mean arterial pressure [MAP], respiratory rate, temperature, and oxygen saturation [SpO_2_]); laboratory parameters such as white blood cell count (WBC), hemoglobin, platelets, glucose, creatinine, blood urea nitrogen (BUN), sodium, potassium, and calcium; and comorbidities including hypertension, myocardial infarction, congestive heart failure, cerebrovascular disease, chronic obstructive pulmonary disease (COPD), liver disease, diabetes, renal disease, malignant cancer, and sepsis. Severity of illness was assessed using the Charlson Comorbidity Index (CCI), Acute Physiology Score III (APS III), Simplified Acute Physiology Score II (SAPS II), Oxford Acute Severity of Illness Score (OASIS), and Sequential Organ Failure Assessment (SOFA) score. Additionally, we extracted first-day ICU interventions, including mechanical ventilation, vasopressor use, and renal replacement therapy (RRT). Comorbidities were identified based on diagnoses recorded at ICU admission. All of the above variables were collected within the first 24 hours of ICU stay and were considered baseline characteristics in this study.

In addition, we extracted vancomycin-related variables, including the cumulative vancomycin dose and TDM records such as trough, peak, and random serum concentrations, all collected during the ICU stay. Vancomycin blood concentrations are recorded in the MIMIC-IV database as part of routine laboratory test results. However, the database does not provide specific information on the analytical methods used to measure these concentrations, such as whether immunoassay, high-performance liquid chromatography (HPLC), or liquid chromatography–mass spectrometry (LC–MS/MS) was employed. Patient outcomes were also collected, with 30-day all-cause mortality defined as the primary endpoint. Detailed information on RRT modalities was obtained from the MIMIC-IV database, where RRT types are categorized as CRRT, continuous venovenous hemofiltration (CVVH), continuous venovenous hemodiafiltration (CVVHDF), continuous venovenous hemodialysis (CVVHD), and slow continuous ultrafiltration (SCUF), as well as IHD and PD. These variables were used to define the primary exposure, classify patients by RRT modality, and evaluate its association with clinical outcomes in this study.

Given the potential influence of calendar time on both RRT practices and TDM implementation, we extracted the anchor year group of each patient from the database and conducted additional analyses, including: (1) the distribution of RRT patients across calendar year subgroups from 2008 to 2022; (2) temporal trends in vancomycin TDM utilization; and (3) multivariable Cox regression analyses (prior to IPTW) stratified by anchor year group to assess the association between TDM and 30-day mortality. The corresponding results are presented in Supplementary Tables S1–S3.

### 2.4 Definitions

The primary outcome of the study was 30-day all-cause mortality, which was defined as death from any cause within 30 days of ICU admission. In this study, vancomycin TDM was defined as the presence of at least one serum drug concentration measurement during the ICU stay, including trough, peak, or random levels. Although TDM can be implemented either through direct measurement of serum concentrations or by estimating AUC, the latter inherently requires one or more measured concentrations (Meng et al., 2019; Drennan et al., 2019; Turner et al., 2018). Therefore, patients with any available vancomycin serum level monitoring were considered to have received TDM, regardless of whether the target was a directly measured trough or an AUC-based estimate (Peng et al., 2024a; Peng et al., 2024b). Accordingly, patients were categorized into the TDM and non-TDM groups based on this definition.

RRT modalities were classified according to the recorded procedure codes in the MIMIC-IV database. Patients who received any form of CRRT, including CVVH, CVVHD, CVVHDF, or SCUF, were grouped into the CRRT category. Patients who received only IHD were classified as the IHD group, while those who received only PD were categorized as the PD group. Patients with unclear or unspecified RRT types were assigned to the unknown group. Those who received two or more distinct RRT modalities, such as CRRT combined with IHD or PD, were categorized as the mixed RRT group.

### 2.5 Statistical analysis

#### 2.5.1 Data preprocessing and descriptive analysis

Before conducting formal statistical analyses, missing values in baseline variables were imputed using the K-nearest neighbors (KNN) algorithm (Faisal and Tutz, 2022). The details of missingness for each variable are presented in Supplementary Table S4. Continuous variables were assessed for normality to determine the appropriate descriptive and inferential statistics. Normally distributed variables were reported as mean ± standard deviation (SD) and compared between groups using Student’s t-test. Non-normally distributed variables were expressed as median and interquartile range [IQR], and the Wilcoxon rank-sum test was used for between-group comparisons. Categorical variables were summarized as counts and percentages (n, %) and compared using either Pearson’s chi-square test or Fisher’s exact test, as appropriate. All statistical tests were two-sided, and a *P*-value <0.05 was considered statistically significant.

#### 2.5.2 Primary outcome and follow-up

The primary outcome of this study was 30-day all-cause mortality. Survival time was calculated from the date of ICU admission to the date of death or the 30th day, whichever occurred first. Patients who survived beyond 30 days were right-censored at day 30.

#### 2.5.3 Propensity score modeling and IPTW weighting

To control for potential selection bias and confounding in treatment assignment, we constructed a multivariable logistic regression model in which receipt of TDM was set as the dependent variable, and baseline clinical characteristics were included as independent variables to estimate each patient’s propensity score (PS). Using the estimated PS, we applied inverse probability of treatment weighting (IPTW), assigning a weight of 1/PS to patients in the TDM group and 1/(1−PS) to those in the non-TDM group. These weights were used to create a weighted pseudo-population in which baseline covariates were balanced between the groups. Covariate balance was evaluated using standardized mean differences (SMDs), with an SMD <0.10 indicating acceptable balance.

#### 2.5.4 Stepwise and weighted cox regression modeling

To assess the impact of confounding on the association between TDM and 30-day all-cause mortality, we developed a series of Cox proportional hazards models with progressively increasing covariate adjustment. These included an unadjusted univariate model and a fully adjusted multivariable model incorporating all baseline variables. To further improve robustness, a doubly robust Cox model was constructed by applying IPTW alongside covariate adjustment. Hazard ratios (HRs) and 95% confidence intervals (CIs) were reported to evaluate the consistency and robustness of the TDM–mortality association across models. Additionally, in both the original and IPTW-weighted populations, Kaplan–Meier survival curves were generated to compare 30-day survival between the TDM and non-TDM groups, and the log-rank test was used to evaluate the statistical significance of survival differences.

#### 2.5.5 Subgroup analysis

Prespecified subgroup analyses were conducted in both the original and IPTW-weighted cohorts. Subgroup variables included age, sex, race, mechanical ventilation, use of vasopressors, receipt of RRT on the first ICU day, severity scores, and RRT modality. To evaluate potential effect modification, interaction terms between TDM and each subgroup variable were incorporated into the Cox regression models. Results were presented as forest plots showing the HRs and 95% CIs for TDM within each subgroup.

#### 2.5.6 Sensitivity analysis

To evaluate potential confounding by treatment intensity, we performed sensitivity analyses using the cumulative vancomycin dose during the ICU stay as a proxy for treatment intensity, on the rationale that larger cumulative doses may indicate prolonged exposure or more aggressive therapy. This variable was included as an additional covariate in an extended multivariable Cox regression model. However, approximately 28% of patients had missing values for this variable. To address potential bias introduced by missingness, we applied three complementary data-handling strategies: (1) Random forest imputation, using the non-parametric missForest algorithm to predict missing values based on all candidate covariates and the outcome, followed by multiple imputation (m = 20) and pooling with Rubin’s rules; (2) Complete case analysis, excluding patients with missing dose data and repeating modeling procedures in the subset of 1,501 patients with complete information; and (3) Missing indicator method, replacing missing values with the cohort median and adding a binary indicator variable (dose_missing) to account for missingness-related prognostic signals. For each approach, we constructed both multivariable Cox regression models and IPTW–adjusted Cox models to reassess the association between TDM and 30-day all-cause mortality. This analytic framework enabled a comprehensive evaluation of the robustness of the estimated treatment effect under varying assumptions regarding missing data.

#### 2.5.7 Software and packages

All statistical analyses were conducted using R software (version 4.5.1). The main R packages used included tableone, survminer, jskm, survival, survey, missForest, forestplot, and dplyr.

## 3 Results

### 3.1 Baseline characteristics

A total of 2,085 critically ill patients receiving RRT were included in this study, with 1,556 patients in the TDM group and 529 in the non-TDM group. Baseline characteristics before and after IPTW are summarized in Table 1. Before weighting, significant baseline imbalances were noted between the two groups. SMDs exceeded 0.10 for several clinical variables, including temperature, glucose, the prevalence of COPD and sepsis, the use of mechanical ventilation and vasopressors, receipt of RRT on the first ICU day, and severity scores such as APS III, OASIS, and SOFA. These imbalances suggest potential confounding effects that may bias the estimated association between TDM and clinical outcomes.

**TABLE 1:** Baseline characteristics of the patients enrolled from the MIMIC-IV database.

Patient characteristic	Before IPTW					After IPTW		
	Total (n = 2085)	Non-TDM group (n = 529)	TDM group (n = 1,556)	P	SMD	Non-TDM group (n = 2,079)	TDM group (n = 2,084)	SMD
Gender [male, n (%)]	1,278 (61.3)	311 (58.8)	967 (62.1)	0.171	0.069	1,260 (60.6)	1276.00 (61.2)	0.013
Age (years)	62.9 ± 15.1	63.6 ± 15.2	62.7 ± 15.1	0.238	0.059	63.2 ± 15.5	63.0 ± 15.1	0.012
Race [white, n (%)]	1,144 (54.9)	275 (52)	869 (55.8)	0.123	0.078	1,129 (54.3)	1,142 (54.8)	0.010
Vital signs								
Heart rate (bpm)	90.5 ± 17.8	90.5 ± 17.2	90.6 ± 18.0	0.898	0.006	90.3 ± 17.4	90.5 ± 18.1	0.012
MAP (mmHg)	74.8 ± 10.5	74.7 ± 10.8	74.8 ± 10.4	0.980	0.001	75.2 ± 11.0	74.8 ± 10.5	0.039
Respiratory rate (/min)	21.4 ± 4.8	21.3 ± 4.9	21.5 ± 4.7	0.393	0.043	21.3 ± 4.8	21.4 ± 4.8	0.020
Temperature (°C)	37.5 ± 0.9	37.4 ± 0.9	37.5 ± 0.9	0.005	0.142	37.5 ± 1.0	37.5 ± 0.9	0.007
Spo2 (%)	96.4 ± 3.3	96.3 ± 3.6	96.4 ± 3.2	0.548	0.029	96.5 ± 3.0	96.4 ± 3.3	0.011
Laboratory tests								
WBC (×10^9^/L)	15.8 (10.9, 22.3)	15.5 (10.4, 22.2)	15.8 (10.9, 22.3)	0.395	0.016	15.0 (9.9, 22.1)	15.8 (10.9, 22.2)	0.048
Hemoglobin (g/L)	9.0 ± 2.2	8.9 ± 2.3	9.1 ± 2.1	0.063	0.092	9.1 ± 2.3	9.0 ± 2.1	0.024
Platelets (×10^9^/L)	129.0 (74.0, 197.0)	127.0 (70.0, 189.0)	130.5 (75.0, 198.0)	0.176	0.074	131.0 (74.4, 198.5)	128.0 (75.0, 197.0)	0.019
Glucose (mg/dL)	156.4 ± 61.1	162.6 ± 66.9	154.4 ± 58.9	0.007	0.131	155.0 ± 59.8	156.1 ± 61.1	0.017
Creatinine (mg/dL)	3.9 (2.4, 6.0)	4.0 (2.4, 6.4)	3.8 (2.4, 5.9)	0.192	0.078	3.9 (2.3, 6.0)	3.9 (2.4, 6.0)	0.001
BUN(mg/dL)	47.0 (31.0, 73.0)	48.0 (29.0, 74.0)	47.0 (31.0, 72.0)	0.266	0.054	47.0 (28.0,72.0)	47.0 (32.0,73.0)	0.019
Sodium (mmol/L)	134.7 ± 5.7	134.9 ± 5.4	134.6 ± 5.8	0.274	0.056	134.9 ± 5.4	134.7 ± 5.8	0.030
Potassium (mmol/L)	5.2 ± 1.1	5.3 ± 1.2	5.2 ± 1.1	0.221	0.061	5.2 ± 1.1	5.2 ± 1.1	0.020
Calcium (mmol/L)	7.8 ± 1.1	7.9 ± 1.0	7.8 ± 1.1	0.143	0.075	7.9 ± 1.0	7.8 ± 1.1	0.032
Comorbidity diseases, n(%)								
Hypertension	1,571 (75.3)	397 (75)	1,174 (75.4)	0.853	0.009	1,568 (75.4)	1,574 (75.5)	0.002
Myocardial infarct	494 (23.7)	127 (24)	367 (23.6)	0.844	0.01	486 (23.4)	493 (23.6)	0.006
Congestive heart failure	858 (41.2)	221 (41.8)	637 (40.9)	0.735	0.017	877 (42.1)	864 (41.5)	0.015
Cerebrovascular disease	255 (12.2)	58 (11)	197 (12.7)	0.304	0.053	242 (11.6)	253 (12.2)	0.016
COPD	544 (26.1)	115 (21.7)	429 (27.6)	0.008	0.136	562 (27.0)	546 (26.2)	0.018
Liver disease	698 (33.5)	167 (31.6)	531 (34.1)	0.282	0.054	659 (31.7)	693 (33.3)	0.033
Diabetes	881 (42.3)	237 (44.8)	644 (41.4)	0.170	0.069	888 (42.7)	880 (42.2)	0.010
Renal disease	1,094 (52.5)	290 (54.8)	804 (51.7)	0.210	0.063	1,129 (54.3)	1,099 (52.7)	0.031
Malignant cancer	234 (11.2)	58 (11)	176 (11.3)	0.827	0.011	215 (10.4)	232 (11.1)	0.024
Sepsis	1,952 (93.6)	468 (88.5)	1,484 (95.4)	<0.001	0.255	1,938 (93.2)	1948 (93.5)	0.013
Severity of illness scores								
CCI	6.9 ± 3.0	7.0 ± 3.0	6.9 ± 3.0	0.712	0.018	6.9 ± 3.1	6.9 ± 2.9	0.003
APS III	87.5 ± 28.8	83.9 ± 28.6	88.8 ± 28.7	<0.001	0.17	86.1 ± 28.6	87.3 ± 28.7	0.044
SAPS II	53.2 ± 15.6	53.5 ± 16.8	53.1 ± 15.1	0.613	0.025	52.3 ± 16.3	53.0 ± 15.2	0.045
OASIS	42.6 ± 10.1	41.0 ± 9.9	43.1 ± 10.1	<0.001	0.208	41.9 ± 9.6	42.5 ± 10.2	0.055
SOFA score	10.1 ± 3.8	9.7 ± 3.8	10.2 ± 3.8	0.011	0.128	10.0 ± 3.8	10.0 ± 3.8	0.035
Therapy, n(%)								
ICU Day 1 RRT	1,198 (57.5)	351 (66.4)	847 (54.4)	<0.001	0.245	1,184 (57.0)	1,194 (57.3)	0.007
Mechanical ventilation	1,220 (58.5)	279 (52.7)	941 (60.5)	0.002	0.156	1,199 (57.7)	1,215 (58.3)	0.013
Vasoactive drug	1,212 (58.1)	250 (47.3)	962 (61.8)	<0.001	0.296	1,187 (57.1)	1,211 (58.1)	0.021

**FIGURE 2:**
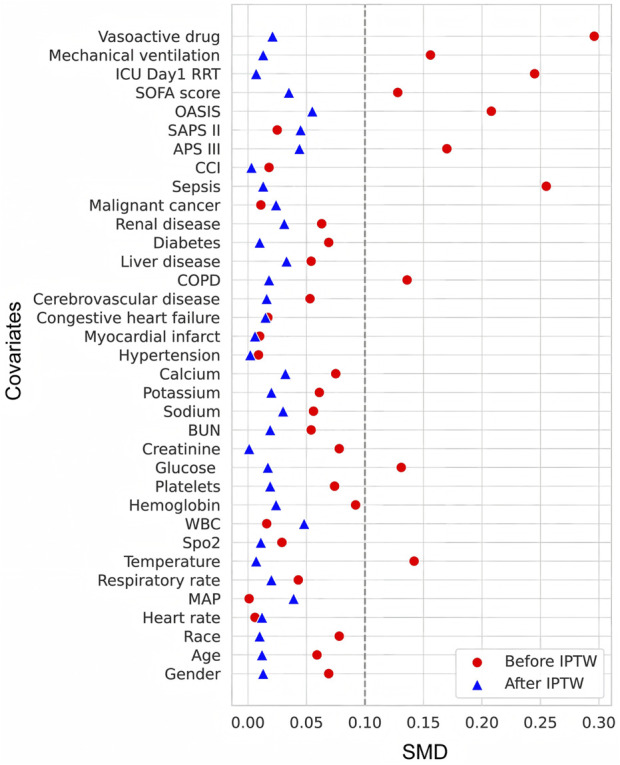
Love plot showing SMDs of covariates before and after IPTW.

Notably, the TDM group comprised approximately 75% of the total sample, resulting in substantial imbalance in group sizes. To minimize bias from this disparity and control for confounding baseline variables, IPTW was employed to construct a weighted pseudo-population. After weighting, the effective sample sizes between the two groups were well balanced, and all covariates achieved SMDs below 0.10 (Figure 2), indicating successful adjustment and a more reliable basis for effect estimation.

### 3.2 Primary outcome

**FIGURE 3:**
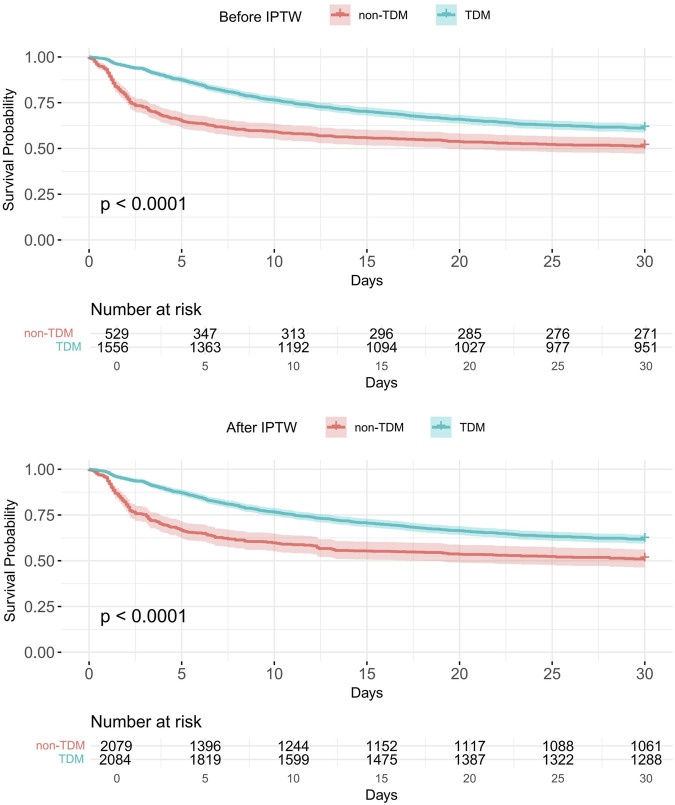
Kaplan–Meier curves for 30-day all-cause mortality according to vancomycin TDM before and after IPTW.

Table 2 presents the association between vancomycin TDM and 30-day all-cause mortality, based on multiple statistical models. Among the 2,085 critically ill patients receiving RRT, the overall 30-day mortality rate was 41.4% (863/2,085), with 38.9% (605/1,556) in the TDM group and 48.8% (258/529) in the non-TDM group. To evaluate the potential impact of confounding, a series of Cox proportional hazards models were constructed with stepwise adjustment for covariates (see Supplementary Table S5 for model details). In the unadjusted model, TDM was significantly associated with reduced 30-day mortality (HR = 0.644, 95% CI: 0.557–0.745, *P* < 0.001). After full adjustment for baseline characteristics listed in Table 1, the association remained robust and was further strengthened (HR = 0.478, 95% CI: 0.409–0.560, *P* < 0.001). To further address potential treatment selection bias, a marginal structural Cox model based on IPTW was applied, yielding consistent findings (HR = 0.645, 95% CI: 0.547–0.761, *P* < 0.001). Additionally, the doubly robust model, which incorporated covariate adjustment on top of IPTW, demonstrated a similar association (HR = 0.457, 95% CI: 0.385–0.544, *P* < 0.001). Kaplan–Meier survival curves before and after IPTW adjustment (Figure 3) further illustrated a significantly higher probability of 30-day survival in the TDM group compared with the non-TDM group.

**TABLE 2:** Association between vancomycin TDM and 30-day mortality: event rates and hazard ratios from different models.

Group/Model	Events/N (%)	HR (95% CI)	P value
All patients	863/2,085 (41.4%)	—	<0.001^a^
Non-TDM group	258/529 (48.8%)	Reference	
TDM group	605/1,556 (38.9%)		
Unadjusted cox model	—	0.644 (0.557∼0.745)	<0.001
Multivariable-adjusted cox model	—	0.478 (0.409∼0.560)	<0.001
IPTW-weighted cox model (marginal model)	—	0.645 (0.547∼0.761)	<0.001
Doubly robust cox model (IPTW + covariates)	—	0.457 (0.385∼0.544)	<0.001

^a^ P-value for difference in 30-day mortality between groups using chi-square test. HRs, were derived from Cox proportional hazards models. The IPTW-weighted model estimates the marginal treatment effect by balancing baseline covariates using inverse probability of treatment weighting. The doubly robust model incorporates both IPTW and additional covariate adjustment.

Collectively, these findings demonstrate a consistent inverse association between TDM and 30-day mortality in critically ill patients undergoing RRT, suggesting that TDM may have clinical relevance in this high-risk population.

### 3.3 Subgroup analysis

**FIGURE 4:**
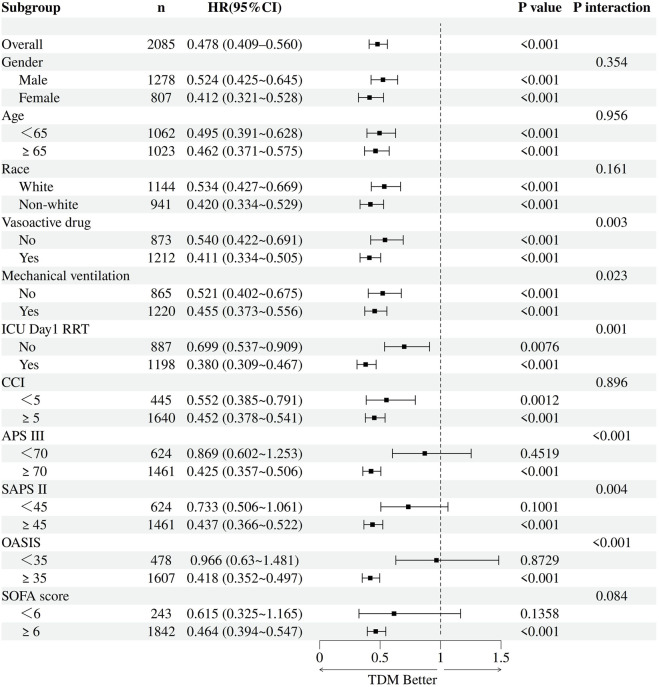
Forest plot of subgroup analyses for the association between vancomycin TDM and 30-day mortality before IPTW.

**FIGURE 5:**
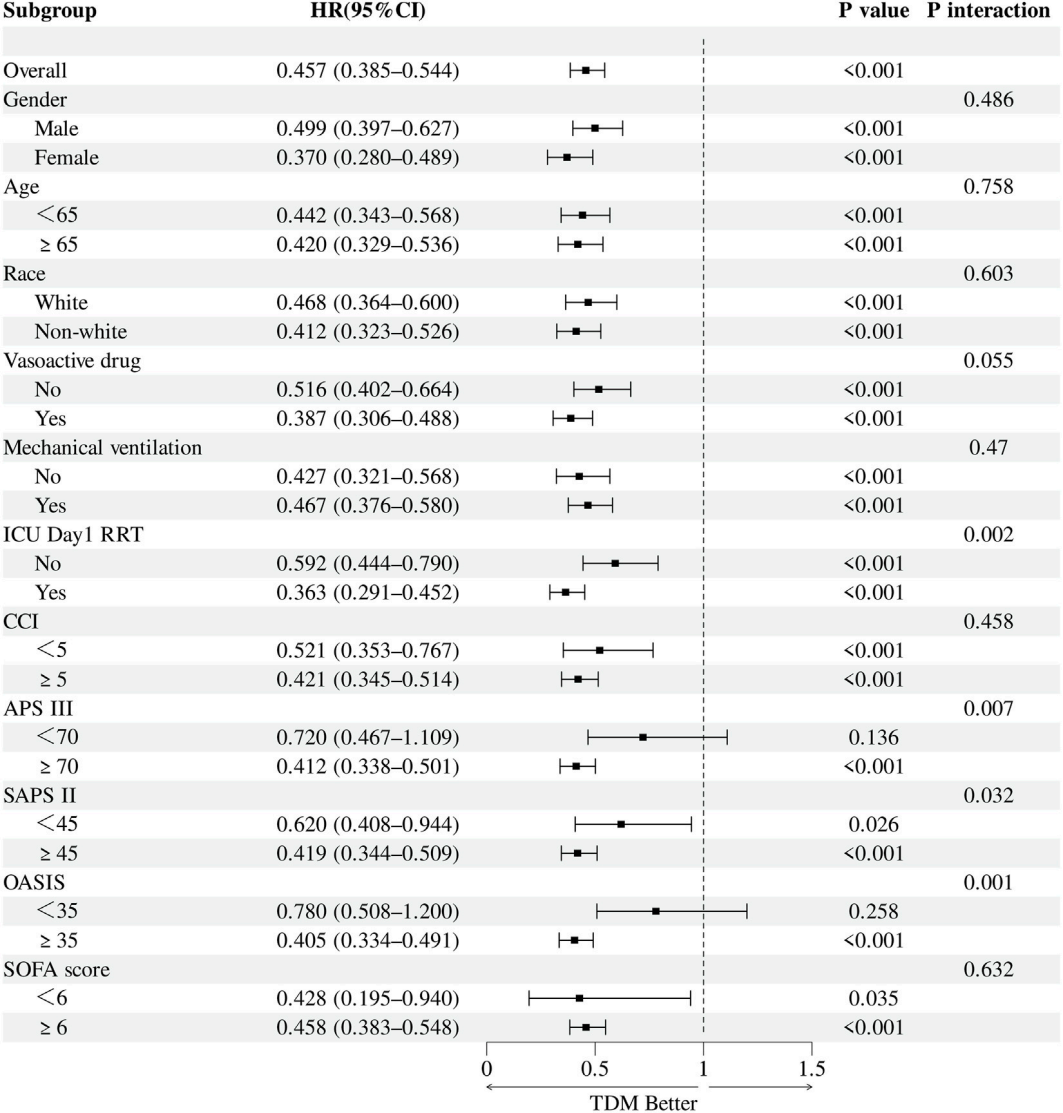
Forest plot of subgroup analyses for the association between vancomycin TDM and 30-day mortality after IPTW.

To further explore potential effect modification, prespecified subgroup analyses were performed following adjustment for confounders. Subgroups were defined by sex, race, age (≥65 vs. <65 years), mechanical ventilation status, vasopressor use, and receipt of RRT on the first ICU day. Additional stratification was conducted based on illness severity using SOFA, APS III, SAPS II, CCI, and OASIS scores. For each subgroup, multivariable and IPTW-weighted Cox regression models were constructed, incorporating interaction terms between TDM and subgroup variables to assess heterogeneity in effect. TDM was robustly associated with reduced 30-day mortality across all strata, irrespective of demographic characteristics, organ support measures, or RRT initiation timing (Figures 4, 5). Notably, the survival benefit of TDM appeared more pronounced in patients with higher severity scores. These findings support the observed association between TDM and reduced mortality in this population.

**FIGURE 6:**
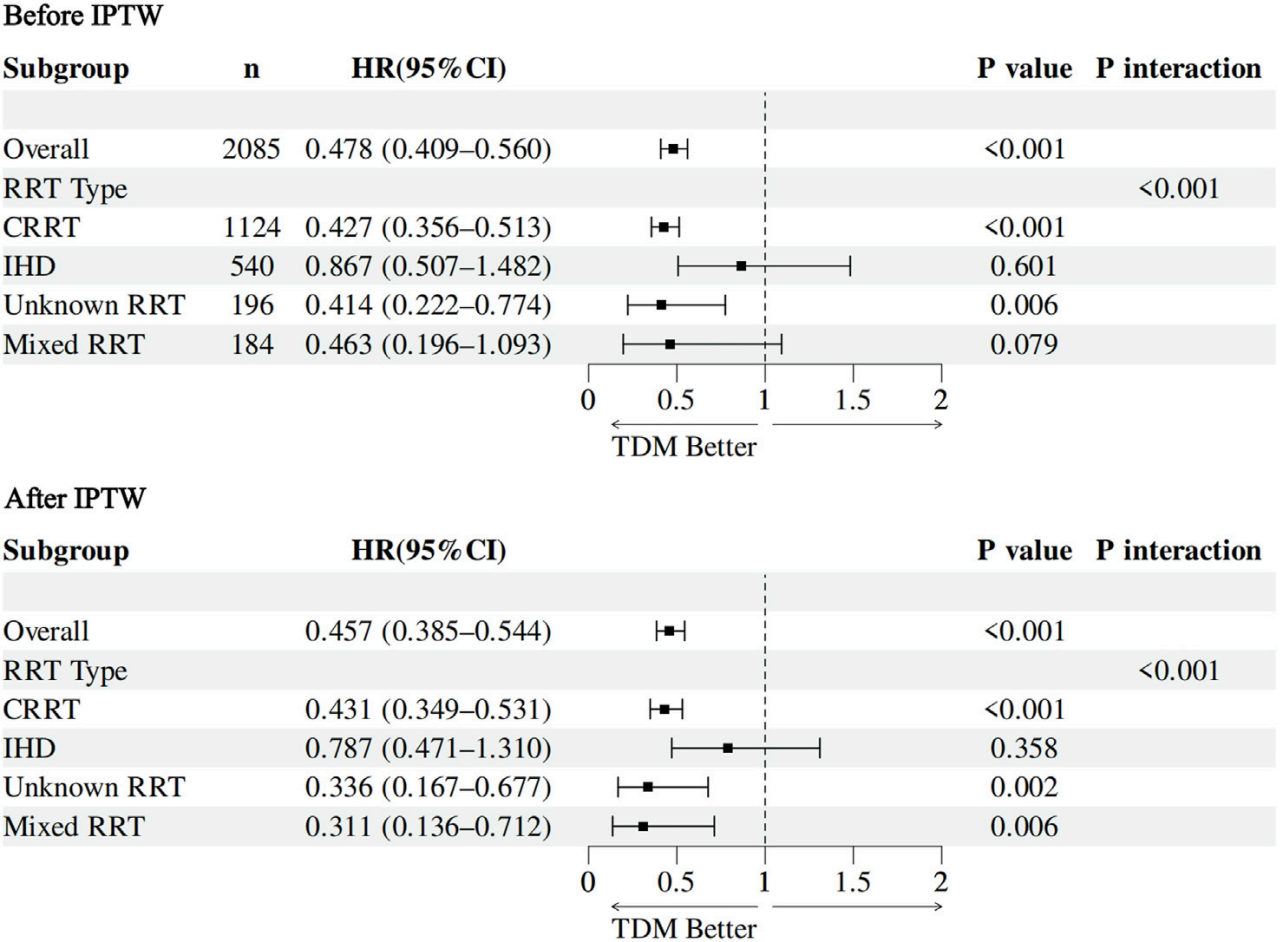
Forest plot of subgroup analyses by RRT modality for the association between vancomycin TDM and 30-day mortality before and after IPTW.

Prespecified stratified analyses were conducted by RRT modality to explore potential heterogeneity in the clinical effectiveness of vancomycin TDM (detailed results are shown in Figure 6). The findings revealed notable differences in the association between TDM and 30-day mortality across RRT types. In patients receiving CRRT, vancomycin TDM was consistently associated with significantly lower 30-day mortality in both multivariable Cox regression and IPTW-adjusted models, with results being stable and robust. For patients treated with IHD, a trend toward reduced mortality was observed in the TDM group; however, the 95% CI included 1.0, indicating the result was not statistically significant. In the PD subgroup, small sample size and low event rates limited the stability of model estimates, precluding definitive conclusions. Among patients with unknown RRT modality, TDM was significantly associated with lower 30-day mortality in both modeling approaches. Furthermore, in patients receiving mixed RRT, the association remained after IPTW adjustment and appear somely stronger. HRs and 95% CIs for each RRT subgroup are presented in Supplementary Table S6. In summary, the protective effect of vancomycin TDM varied across RRT modalities, with the strongest benefit observed among CRRT recipients.

### 3.4 Sensitivity analysis

To assess the potential influence of vancomycin treatment intensity on 30-day mortality and its impact on the estimated effect of TDM, a sensitivity analysis was performed. While longer treatment duration or higher cumulative doses may correlate with improved outcomes, substantial missing data were present for both variables due to limitations of the MIMIC database. In particular, treatment duration may be affected by inaccuracies in recorded medication order timestamps. Therefore, cumulative vancomycin dose during the ICU stay was selected as a proxy measure for treatment intensity.

As shown in Table 3, TDM remained significantly associated with reduced 30-day all-cause mortality across all three missing data handling strategies and both analytical models (HR range: 0.474–0.610; all *P* < 0.001). These results support the robustness of the inverse association between TDM and mortality across different missing data handling approaches. The observed association between TDM and reduced mortality was robust across different patterns of dose data missingness and imputation strategies, suggesting that TDM may be independently associated with improved survival, beyond treatment duration or dosing intensity.

**TABLE 3:** Sensitivity analysis of the association between vancomycin TDM and 30-day mortality using three strategies for handling missing cumulative dose data.

Missing data handling method	Analysis model	HR (95% CI)	P value
Random forest imputation	Multivariable-adjusted cox model	0.582 (0.492–0.688)	<0.001
	Doubly robust model (IPTW + covariates)	0.610 (0.507–0.734)	<0.001
Complete case analysis	Multivariable-adjusted cox model	0.544 (0.448–0.660)	<0.001
	Doubly robust model (IPTW + covariates)	0.474 (0.380–0.591)	<0.001
Missing-indicator method	Multivariable-adjusted cox model	0.562 (0.477–0.663)	<0.001
	Doubly robust model (IPTW + covariates)	0.493 (0.393–0.618)	<0.001

## 4 Discussion

### 4.1 Main findings

In this retrospective cohort study using data from the MIMIC-IV database, we systematically evaluated the association between vancomycin TDM and 30-day all-cause mortality in critically ill patients receiving RRT. After adjusting for potential confounders using multivariable Cox regression, IPTW, and doubly robust models, TDM was consistently associated with lower 30-day mortality. Kaplan–Meier analysis suggested a higher probability of survival in the TDM group, with the strongest inverse association observed in patients undergoing CRRT. These findings suggest that vancomycin TDM may be associated with improved short-term survival outcomes in this high-risk population, although prospective studies are warranted to confirm these results.

### 4.2 Potential mechanisms

The observed survival benefit of vancomycin TDM in patients receiving RRT may involve several underlying mechanisms acting in synergy. First, vancomycin clearance in RRT patients is influenced by multiple factors, including dialysis modality, dose, membrane permeability, and residual renal function, leading to significant fluctuations in drug concentrations (Lakshmipathy et al., 2024; Joy et al., 1998; Pistolesi et al., 2019; Thompson, 2008). Empirical dosing often fails to maintain drug exposure within the therapeutic window (Alshehri et al., 2020; Obara et al., 2016). TDM provides real-time pharmacokinetic feedback to guide individualized dose adjustments, thereby helping maintain safe and effective serum levels (Rybak et al., 2020; Smeets et al., 2023). Second, TDM facilitates the early detection of elevated trough concentrations, enabling timely interventions to reduce the risk of nephrotoxicity and ototoxicity, which may otherwise contribute to complications and increased mortality (Lim et al., 2023; Ishigo et al., 2024). Third, due to dynamic changes in dialysis parameters and patient status, drug clearance is often highly variable; regular TDM allows timely identification and correction of abnormal exposure (Kanji et al., 2023). Lastly, achieving stable and adequate antimicrobial exposure may improve infection control and clinical efficacy (Kullar et al., 2011; Holmes et al., 2013). When integrated into multidisciplinary care, TDM can further support optimization of both antimicrobial therapy and dialysis strategies. However, it should be noted that the clinical value of TDM depends on the appropriate use of vancomycin as part of a rational therapeutic plan.

Notably, this study found that vancomycin TDM was significantly associated with reduced mortality among patients receiving CRRT, whereas only a non-significant trend toward improved survival was observed in those undergoing IHD. This discrepancy may reflect underlying differences in pharmacokinetic variability and clinical context between the two modalities. In CRRT, vancomycin clearance is influenced by continuously fluctuating dialysis parameters, membrane properties, and patient-specific factors, making drug exposure difficult to predict and may necessitate frequent dose adjustments (Smeets et al., 2023; Kirwan et al., 2021; Yu et al., 2023). TDM may play an important role in such settings by providing real-time feedback to guide individualized dosing. By contrast, IHD involves scheduled drug removal, and vancomycin levels tend to be more stable and predictable during interdialytic intervals, potentially limiting the additional value of TDM(Lewis and Nolin, 2021; Zamoner et al., 2024). Furthermore, patients receiving CRRT are often more critically ill, and the adequacy of antimicrobial exposure may be more closely associated with short-term outcomes. In IHD patients, who are generally more stable, mortality is likely influenced by a broader range of factors, which may attenuate the observable benefit of TDM. It is also possible that the smaller sample size and event count in the IHD subgroup limited statistical power to detect a significant association. Together, these findings suggest that the clinical value of TDM may vary by RRT modality, with the most pronounced benefit observed in CRRT patients.

### 4.3 Comparison with previous studies

Contemporary research on vancomycin use in patients undergoing RRT primarily follows two interrelated directions. The first focuses on characterizing the PK/PD profiles of vancomycin across different RRT modalities using population pharmacokinetic models combined with Bayesian TDM (Oda et al., 2020). These studies aim to identify key covariates influencing vancomycin clearance under various RRT settings (Joy et al., 1998; Yu et al., 2023; Udy et al., 2013). For instance, in patients receiving CRRT, drug clearance is largely determined by the effluent rate—the combined flow of dialysate and replacement fluids—with body weight and residual renal function also contributing significantly (Wang et al., 2023). Building on these mechanistic insights, a second major line of investigation has proposed stratified dosing strategies tailored to RRT-specific PK characteristics (Kirwan et al., 2021; Charoensareerat et al., 2019). In conventional-intensity CRRT (effluent rate approximately 20–25 mL/kg/h, comparable to a creatinine clearance of 30–50 mL/min), guidelines typically recommend an initial loading dose of 20–25 mg/kg followed by maintenance dosing of 7.5–10 mg/kg every 12 h, with Bayesian AUC-guided TDM conducted within the first 24 h (Rybak et al., 2020). For patients undergoing high-intensity CRRT (e.g., dialysate flow rate >30 mL/kg/h), retrospective studies suggest that daily doses ≥15 mg/kg are generally required to achieve therapeutic targets promptly (Srour et al., 2023). In patients undergoing IHD, real-time AUC monitoring is often impractical (Lewis and Nolin, 2021). Therefore, clinical guidelines typically recommend targeting pre-dialysis trough concentrations of 15–20 mg/L as a feasible surrogate marker. Post-dialysis supplemental dosing is then administered, with further individualization based on factors such as body weight, dialyzer membrane permeability, timing of administration relative to dialysis, and drug loss during the dialysis session (Rybak et al., 2020; Lewis and Nolin, 2021; Rybak et al., 2020). For patients undergoing PD, particularly in the treatment of peritonitis, the 2022 guidelines of the International Society for Peritoneal Dialysis (ISPD) recommend intraperitoneal administration of vancomycin as the preferred route. The guidelines also emphasize that each dwell should last at least 4 h to ensure adequate peritoneal exposure and systemic absorption. However, recent study has indicated that the current guideline-recommended regimens may result in subtherapeutic drug exposure in a significant number of patients, posing a risk of underdosing (Li et al., 2022). To mitigate this issue, some researchers have proposed avoiding intermittent intraperitoneal administration and instead adopting a continuous dosing strategy, starting with a loading dose of 20 mg/kg followed by maintenance dosing with 50 mg/L of vancomycin per dwell, to optimize intraperitoneal drug exposure.

In summary, prior research has established a well-characterized pharmacokinetic/pharmacodynamic (PK/PD) foundation for vancomycin therapy in patients receiving RRT, and has led to the development of stratified dosing strategies (Claisse et al., 2019). These efforts underpin a treatment paradigm based on “modeling–monitoring–optimization.” However, high-quality prospective evidence remains lacking regarding whether the implementation of TDM or the use of different dosing strategies translates into improved clinical outcomes such as survival (Stewart et al., 2021; Corrêa et al., 2025). Most existing studies focus on surrogate endpoints, such as target attainment or nephrotoxicity, and are limited by significant heterogeneity in RRT prescriptions and inadequate control of confounding factors—posing challenges for extrapolating clinical benefit (Roberts et al., 2021). Therefore, systematic research focusing on hard clinical endpoints is warranted to address current evidence gap and inform optimization of vancomycin use in this high-risk population.

### 4.4 Strengths of the study

This study employed multiple statistical methods and sensitivity analyses to ensure the robustness of the findings and offer clinically relevant insights. Methodologically, multivariable regression, IPTW, and doubly robust models were applied to address confounding and achieve covariate balance. Missing data on cumulative vancomycin dose were handled using three different approaches, all yielding consistent results that supported the robustness of the primary findings. Additionally, the study systematically compared the effects of TDM across different RRT modalities within a single cohort, providing evidence to support individualized treatment strategies. The large sample size, extended study period (2008–2022), and diverse patient population enhance the generalizability of the results, although further external validation is warranted. Overall, our findings suggest that standardized TDM may help guide clinical decision-making in high-risk RRT populations and support optimization of vancomycin therapy.

### 4.5 Limitations

This study has several limitations. First, although various statistical approaches were applied to adjust for confounding, the possibility of residual confounding inherent in observational designs cannot be completely excluded. Second, as the database recorded only serum drug concentrations and lacked AUC data, comparisons between trough-based and AUC-guided monitoring strategies could not be performed. Third, the analysis was based on data from a single center, and center-specific RRT protocols may limit the external validity of the findings. Fourth, this study only assessed 30-day all-cause mortality as a short-term outcome, without addressing long-term prognosis or economic outcomes. In addition, the decision to perform TDM was not randomized and may have been influenced by clinician preference, illness severity, or resource availability, potentially introducing indication bias despite statistical adjustments. Finally, although an inverse association between TDM and mortality was observed, causal inferences should be made cautiously in the absence of interventional evidence.

## 5 Conclusion

This study found that among critically ill patients receiving RRT, vancomycin TDM was significantly associated with reduced 30-day all-cause mortality, particularly in those CRRT. These findings suggest that TDM may help optimize antimicrobial therapy and improve short-term outcomes in high-risk RRT populations.

## Data Availability

The original contributions presented in the study are included in the article/Supplementary Material, further inquiries can be directed to the corresponding authors.
