# Supplementary material for: Association between vancomycin therapeutic drug monitoring and improved clinical outcomes in critically ill patients receiving renal replacement therapy: a retrospective cohort study
Source: Front Pharmacol. 2026 Jan 7;16:1715023. doi: 10.3389/fphar.2025.1715023 (PMC12819319; doi:10.3389/fphar.2025.1715023)
Supplement: Supplementary file 1 [file Supplementaryfile1.pdf]

Supplementary Material

Supplementary Table 1： Distribution of included patients across anchor year groups (2008 – 2022)

Anchor Year Group	Count	Percentage
2008 - 2010	502	24.1%
2011 - 2013	360	17.3%
2014 - 2016	380	18.2%
2017 - 2019	449	21.5%
2020 - 2022	394	18.9%
**Total**	**2085**	**100.0%**

Supplementary Table 2： Trends in Vancomycin TDM utilization across anchor year groups

Variables	Total (n = 2085)	2008 - 2010 (n = 502)	2011 - 2013 (n = 360)	2014 - 2016 (n = 380)	2017 - 2019 (n = 449)	2020 - 2022 (n = 394)	*p*
**vanTDM, n (%)**							< 0.001
**non-TDM**	529 (25.4)	107 (21.3)	81 (22.5)	92 (24.2)	95 (21.2)	154 (39.1)	
**TDM**	1556 (74.6)	395 (78.7)	279 (77.5)	288 (75.8)	354 (78.8)	240 (60.9)	

Supplementary Table 3： Subgroup Analysis of 30-Day Mortality in RRT Patients With or Without Vancomycin TDM Across Anchor Year Groups (2008 – 2022) Using Multivariable Cox Regression before IPTW

Subgroup	Variable	n.total	n.event_%	crude.HR_95CI	crude.P_value	adj.HR_95CI	adj.P_value	P.for.interaction
								0.691
**2008 - 2010**	non-TDM	107	37 (34.6)	1(Ref)		1(Ref)		
	TDM	395	124 (31.4)	0.81 (0.56~1.17)	0.257	0.57 (0.38~0.84)	0.005	
**2011 - 2013**	non-TDM	81	36 (44.4)	1(Ref)		1(Ref)		
	TDM	279	96 (34.4)	0.63 (0.43~0.92)	0.018	0.35 (0.22~0.55)	<0.001	
**2014 - 2016**	non-TDM	92	45 (48.9)	1(Ref)		1(Ref)		
	TDM	288	107 (37.2)	0.59 (0.42~0.84)	0.003	0.35 (0.23~0.53)	<0.001	
**2017 - 2019**	non-TDM	95	49 (51.6)	1(Ref)		1(Ref)		
	TDM	354	165 (46.6)	0.73 (0.53~1)	0.051	0.41 (0.29~0.6)	<0.001	
**2020 - 2022**	non-TDM	154	91 (59.1)	1(Ref)		1(Ref)		
	TDM	240	113 (47.1)	0.62 (0.47~0.82)	0.001	0.54 (0.4~0.74)	<0.001	

Supplementary Table 4：detailed information on missing data of variables in the study

variable	inventory	N missing	missing rate
Heart rate	2082	3	0.14%
MAP	2082	3	0.14%
Respiratory rate	2082	3	0.14%
Temperature	1990	95	4.56%
Spo2	2081	4	0.19%
WBC	2079	6	0.29%
Hemoglobin	2078	7	0.34%
Platelets	2080	5	0.24%
Creatinine	2080	5	0.24%
BUN	2079	6	0.29%
Glucose	2077	8	0.38%
Calcium	2039	46	2.21%
Sodium	2080	5	0.24%
ICU cumulative vancomycin dose	1501	584	28.01%

The other variables in this study do not missing.

Supplementary Table 5：Association between vancomycin TDM and 30-day mortality in critically ill patients receiving RRT before and after IPTW with different sets of covariates

	Before IPTW			After IPTW		
	**HR[a]**	**95% CI**	**p-value**	**HR[b]**	**95% CI**	**p-value**
Model 1	0.644	(0.557~0.745)	＜0.001	0.645	0.547 - 0.761	＜0.001
Model 2	0.651	(0.563~0.754)	＜0.001	0.646	0.548 - 0.761	＜0.001
Model 3	0.551	(0.474~0.639)	＜0.001	0.529	0.446 - 0.627	＜0.001
Model 4	0.516	(0.444~0.601)	0.001	0.492	0.415–0.584	＜0.001
Model 5	0.473	(0.405~0.553)	＜0.001	0.462	0.387 - 0.550	＜0.001
Model 6	0.478	(0.409~0.56)	＜0.001	0.457	0.385 - 0.544	＜0.001

[a]Hazard ratios (HRs) derived from Cox proportional hazards regression models adjusted for covariates; [b]Hazard ratios (HRs) derived from IPTW-weighted multivariable Cox proportional hazards regression models adjusted for covariates. Adjusted covariates: Model 1 = Vancomycin TDM only; Model 2 = Model 1+(Gender, Age, Race); Model 3 = Model 2+(Heart rate , MAP, Respiratory rate, Temperature, and SpO2)+(WBC, Hemoglobin, Platelets, Creatinine, BUN, Glucose, Sodium, Potassium, and Calcium); Model 4 = Model 3+(Hypertension, Myocardial infarct, Congestive heart failure, Cerebrovascular disease, COPD, Liver disease, Diabetes, Renal disease, Malignant cancer, and Sepsis); Model 5 = Model 4+(CCI, SOFA score, APS III, SAPS II, OASIS); Model 6 = Model 5+(ICU Day1 RRT, Vasoactive drug, Mechanical ventilation).

Supplementary Table 6: Association between vancomycin TDM and 30-day mortality in critically ill patients receiving different RRT modalities before and after IPTW

RRT modality (n)	Crude HR (95% CI, p)	Multivariable-adjusted Cox model HR (95% CI, p)	IPTW univariable HR (95% CI, p)	Doubly robust model (IPTW + covariates) HR (95% CI, p)
CRRT (n=1124)	0.497 (0.420–0.589), <0.001	0.427 (0.356–0.513), <0.001	0.589 (0.481–0.722), <0.001	0.431 (0.349–0.531), <0.001
IHD (n=540)	1.257 (0.763–2.071), 0.369	0.867 (0.507–1.482), 0.601	1.040 (0.611–1.780), 0.880	0.787 (0.471–1.310), 0.358
PD (n=41)	1.082 (0.258–4.530), 0.914	—	1.170 (0.254–5.390), 0.840	—
Unknown type (n=196)	0.781 (0.487–1.253), 0.306	0.414 (0.222–0.774), 0.006	0.739 (0.442–1.230), 0.247	0.336 (0.167–0.677), 0.002
Mixed RRT (n=184)	0.824 (0.423–1.604), 0.569	0.463 (0.196–1.093), 0.079	0.754 (0.370–1.540), 0.438	0.311 (0.136–0.712), 0.006
